# European Union and its progress towards affordable and clean energy in context of the Agenda 2030

**DOI:** 10.1371/journal.pone.0291933

**Published:** 2023-12-21

**Authors:** Silvia Megyesiova, Emília Dul’ová Spišáková, Barbora Gontkovičová

**Affiliations:** 1 Department of Quantitative Methods, Faculty of Business Economics with seat in Košice, University of Economics in Bratislava, Bratislava, Slovakia; 2 Department of Economics and Management, Faculty of Business Economics with seat in Košice, University of Economics in Bratislava, Bratislava, Slovakia; Università degli Studi di Bari Aldo Moro: Universita degli Studi di Bari Aldo Moro, ITALY

## Abstract

The topic related to affordable and clean energy is currently highly actual. It is essential to realize that affordable and clean energy is energy without negative effects on the environment. Its advantage is that a lot of clean energy is renewable. Therefore, this type of energy contributes positively to the development of several spheres in the economy, such as agriculture, trade, communications, education, health and transport. The aim of the study is to analyze changes in the development of sustainable indicators set of affordable and clean energy in relation to Sustainable Development Goal 7 in the European Union, using cluster analysis to identify the differences and compare changes in the grouping of countries into clusters in the two years studied (2010, 2020). In addition to categorizing countries and confirming differences among member states of the European Union, the results allow us to evaluate the contribution of selected indicators to achieving affordable and clean energy. Our findings indicate that primary energy consumption per capita, final energy consumption in households per capita, energy productivity, share of renewable energy in gross final energy consumption and population unable to keep their home adequately warm shows a positive trend and supports the achievement of the Sustainable Development Goal 7.the energy import dependency is a problematic area in several countries. The results of cluster analysis showed that the largest shifts within the clusters were recorded in Italy, Estonia, and Luxembourg. The most positive shift occurred in Italy due to a significant improvement in four indicators. Luxembourg as a solo country cluster showed in 2020 some of the worst results due to the highest primary energy consumption per capita and the lowest share of renewable energy in gross final energy consumption. The dynamics of Estonia within the clusters was marked by the lowest value of the indicator of dependence on energy imports, as well as an increase in the share of renewable energy and insufficient energy productivity. The results of our study also confirm, that Sweden and Finland are leader countries, which despite high energy consumption are applying renewable energy sources to a greater extent by which they make a positive movement toward affordable and clean energy.

## Introduction

In order to achieve a long-term positive effect of the development of economies, its sustainability must be ensured. It means not to base current development on high current consumption, but to support the future growth of economies by leaving resources to meet the needs of future generations. The United Nations understand sustainable development as development aimed at eliminating poverty, inequalities and promoting sustainable management of natural resources and ecosystems. It is about sustainable, inclusive and fair economic growth, which are the basic prerequisites for increasing the quality of life of both individuals and society as a whole [1].

Sustainable development cannot be defined by one definition. Over the past thirty years, this issue has received significantly greater emphasis and has increasingly included interconnections between natural and human systems, including attention to issues of environmental justice [2]. In this sense, the economic, social and environmental pillars of sustainable development have been widely accepted in the literature and in international organizations since the beginning of this century. Current studies are already aware that economic development negatively affects the ecological footprint [3–5] and the issue of the sustainability of economic development and the energy goals related to it, are solved by several strategies.

The activities of the EU in the field of a climate change and renewable energy have a long-term nature. Since 2010, the Europe 2020 strategy was in force, within which it was one of the three priorities aimed at sustainable growth linked to the support of a greener and more competitive economy that uses resources more efficiently [6]. In addition, this strategy focused on achieving inclusive and smart growth, through the definition of targets in five main areas that the European Union (EU) should achieve by 2020. These targets were in the field of research and development, education, employment, poverty and also environment.

In 2015, the Paris Agreement was adopted as a universal agreement on climate change, which was ratified and signed by all EU member states. In the same year, the United Nations created a new strategy Agenda 2030 for Sustainable Development. The environmental area is covered by two sustainable development goals (SDG)–SDG 7 "Affordable and clean energy" and SDG 13 "Climate action" [7].

Analysis of the current state and repeated control of the achievement of goals in the monitored area is a necessity to achieve the final goals set for the year 2030, respectively for a later period. The set of targets and indicators of the Europe 2020 strategy corresponds to the logic of the Agenda 2030 and the goals of sustainable development [8], which enables the evaluation of the achievement of the targets of the Europe 2020 strategy in the context of the Agenda 2030.

The study deals only with the area of affordable and clean energy, therefore it only takes into account SDG 7. This goal points to the necessity of ensuring universal access to affordable, reliable and sustainable energy. Monitoring SDG 7 in the EU context includes monitoring the evolution of energy consumption, energy supply and access to available energy [9].

The aim of the study is to analyse changes in the development of sustainable indicators set for affordable and clean energy in relation to SDG 7 in the EU, using cluster analysis to identify the differences and compare changes in the grouping of countries into clusters in the two monitored years (2010 and 2020).

The basic difference of this study compared to several others is in the selection of indicators and the determination of the time period for their comparison. SDGs of the 2030 Agenda are set at the global level. However, the natural disparity of the continents requires an individual approach in setting indicators, which Eurostat is also aware of. Eurostat has identified 100 indicators more relevant for European policies. This approach made it possible to define the indicators used for the Europe 2020 strategy and for monitoring some SDGs from the 2030 Agenda. Specifically, the SDG 7 corresponds to the goals of the Europe 2020 strategy in the area of energy and climate.

The agreement of several indicators enables monitoring of the long-term trend of development. This fact gives us the opportunity to assess SDG 7 on the basis of the indicators set by the EU, even at a time when Agenda 2030 did not yet exist. The mentioned approach allows us to assess the progress or stagnation of EU countries in the environmental field in the long-term.

The main motivation of the study is to look for common features and differences between EU countries and, through a time comparison, point out their progress in the area of energy consumption and supply, as well as access to available energy. It will enable us to perform unidimensional and multidimensional analysis of monitored indicators, analysis of time series, as well as identification of associations between selected indicators. The achieved results will allow us to identify the relationships between the selected indicators, as well as the strengths and weaknesses of the countries. The motivation is also to share the achieved results not only with the scientific community, but also with the general public.

## Theoretical framework

According to the of the European Commission, the SDGs have become an important and integral part of the political agenda. They play an important role in the political planning of several activities both inside each country and in relation to the rest of the world. The importance of the orientation of the political system to the SDGs and the achievement of the target values has increased substantially due to the pandemic caused by COVID-19, which can contribute to a more inclusive, sustainable, fairer and more resilient development of countries in the coming years [10]. According to 2030 Agenda as well as the strategy Energy Union, the countries’ orientation towards renewable energy sources and the related increase in energy efficiency represent the basis for the long-term fight against climate change and the improvement of energy security. In this context, the Agenda 2030 can help countries supplement the commitment resulting from the Paris Agreement, to which all EU member states are signatories.

The essence of SDG 7 is to ensure access to clean and affordable energy, which is crucial for the development of agriculture, trade, communications, education, health and transport. Inadequate access to energy hinders economic and human development [1]. Clean energy is energy that comes from renewable sources with zero emissions that do not pollute the air when used. This includes energy saved by energy efficiency measures [11].

There is a certain degree of overlap between clean energy and green, or renewable energy sources. Clean energy works by producing energy without negative impacts on the environment. This is, for example, the release of greenhouse gases such as carbon dioxide. Green energy is energy obtained from natural sources. There is a slight difference between these two types of energy [11]. A lot of clean energy is also renewable. Renewable energy is energy produced from sources that are constantly replenished [12]. Unlike fossil fuels and gas, these renewable energy sources do not run out and include wind and solar energy.

Clean energy provides a number of environmental and economic benefits, including reductions in air pollution. Since renewable energy sources do not emit greenhouse gases such as carbon dioxide, they do not contribute to global warming [13]. These renewables mean that climate change is not progressing, while measures such as reforestation can help mitigate the climate damage already done while reducing global warming. Diverse clean energy supplies also reduce dependence on imported fuels and the associated financial and environmental costs. Renewable clean energy also has inherent cost savings as there is no need to extract and transport fuels such as oil or coal, because the resources are naturally replenished [14]. Other industrial benefits of the clean energy mix are the creation of jobs to develop, manufacture and install the clean energy sources of the future [15].

As the world’s population grows over the long term, the demand for energy is constantly increasing, and renewable sources are the answer to provide sustainable energy solutions while protecting the planet from climate change [12]. Of course, given that fossil fuels are a limited resource, it makes sense that the future is renewable, and thus it is expected that the number of renewable sources will continue to grow.

The issue of affordable and clean energy was and still is the subject of several researches that emphasize the need to make efforts, to bring about changes and innovations that are necessary for achieving long-term goals in the environmental field. Despite the common environmental policy, which is the priority of the European Union for the next few years, studies identify differences between countries. Based on their results, we can identify the leaders and then point out the effectiveness of the tools used to achieve the climate and energy goals in these countries.

A study by Ligus and Peternek [16] and Tutak et al. [17] showed that in the period 2017–2018, countries such as Denmark, Luxembourg, Austria and Sweden were included in the group of so-called “leaders” in the field of energy sustainability. The results of these studies confirmed that Sweden was the leader in the monitored area. Among the countries that diverged the most from the set targets were Belgium, Cyprus and Luxembourg.

A study by Firoiu et al. [18] was focused on how the member states of the EU were able to implement SDG 7 during a five-year period after the acceptance of the Paris Agreement. The main method used was hierarchical cluster analysis, the aim of which was to reveal hidden associative structures. The results of the cluster analysis divided the countries into clusters with high-performing countries and to clusters that do not reach the required values of the monitored indicators in the area of the transition to a greener economy.

The necessity of focusing on climate changes, energy and achieving goals in this area is also supported by the opinions of Chirambo [19] and Che et al. [20], according to which any improvements in this area have a positive effect on several other indicators, such as industrialization, poverty or even the ability of regions to adapt more easily to climate change.

Quito et al. [21] concluded that the use of renewable energies supports the improvement of energy efficiency, and also that replacing the use of traditional energies with alternative sources has a positive impact on national energy efficiency.

According to Širá et al. [22], the Baltic states, the Nordic countries (members of the European Union), Romania and Croatia are among the countries that best meet the targets of the sustainable growth. Even the Croatia was the only country that achieved the targets during the entire period under review.

Siksnelyte-Butkiene et al. [23] elaborated a study according to which countries such as Greece, Croatia, Italy, Portugal and Romania have high potential in the area of reducing greenhouse gas emissions, but also in the area of using renewable energy sources and increasing energy efficiency. They achieved these results thanks to the implementation of effective policies and the adoption of measures leading to success in the monitored area. They also state that the lagging countries include France, Belgium, Ireland, and Poland, which face greater difficulties in implementing climate and energy targets. Their problem is insufficient reduction of greenhouse gas emissions, but also other circumstances related to dependence on fossil fuels (for example, coal in Poland, oil and natural gas in Belgium) and in France (nuclear energy, oil and gas).

## Materials and methods

Sustainability and development are the key issues of countries all over the world. The countries of the EU focus their activities and effort to creating a more sustainable, better and safer world for all [24–27].

The condition and progress towards the “Sustainable Development Goal 7 –Affordable and clean energy” is in the EU measured using a set of seven indicators [24, 25], namely: primary energy consumption, final energy consumption, final energy consumption in households per capita, energy productivity, share of renewable energy in gross final energy consumption, energy import dependency, population unable to keep home adequately warm. For analytical purposes, the analysis of state and development of the EU countries regarding the SDG 7 variables was done using six variables that were downloaded from the Eurostat database. As the final energy consumption and the final energy consumption in households per capita are strongly associated, only one of these indicators was used for analytical purposes. To be able to follow the direction of change, the analysis starts in 2010. This initial position was then compared with the situation in 2020, which was the most recent year according to the availability of the dataset. The variables are either stimulants or destimulants. An indicator is a stimulant if its maximization is considered positively while a variable is a destimulant if its minimalization is considered positively. From the total collection of six indicators, two are stimulants and four are destimulants. As the energy consumption can be associated with some conditions of a specific country, in order to create an overall picture, two more variables were chosen for the analysis: the average temperature and the share of the gross value added (GVA) in industry as a percentage of the total GVA to complete the indicators`collection. The last two variables are neither stimulants nor destimulants, but can be important in discovering the overall reflection in state and development of sustainable and affordable energy consumption, production or productivity.

Based on the stated facts, the following eight indicators were included in the study [7, 24, 25, 28]:

x_1_ primary energy consumption per capita, in tonnes of oil equivalent (TOE),
x_2_ final energy consumption in households per capita, in kilograms of oil equivalent (KGOE),
x_3_ energy productivity, in purchasing power standard (PPS) per kilogram of oil equivalent,
x_4_ share of renewable energy in gross final energy consumption, percentage of total,
x_5_ energy import dependency, percentage of total,
x_6_ population unable to keep home adequately warm, percentage of total,
x_7_ temperature °C, average for the time span between 1991–2020,
x_8_ GVA in industry (except construction), percent of total, average for the period 2010–2020.

The indicator x_1_ counts for the country´s total energy demand, excluding all non-energy use of energy carries. This indicator covers the energy consumption by end users (industry, transport, household, services and agriculture) plus energy consumption of the energy sector itself for the production and transformation of energies, losses during transportation and distribution of energies. The primary energy consumption is calculated per habitant in tonnes of oil equivalent. On the other hand, the final energy consumption measures the energy end-use in a country, excluding all non-energy use of energy carriers. The indicator x_2_ counts for the electricity and heat every citizen consumes at home excluding energy used for transportation and is calculated per capita in kilograms of oil equivalent. The variable x_3_ represents the amount of economic output that is produced per unit of gross available energy. The gross available energy means the total energy demand, i.e., the quantity of energy products needed to satisfy all demand of entities in an EU country and the economic output is given in the unit of purchasing power standard. The indicator x_4_ is defined as the share of renewable energy consumption in gross final energy consumption according to the Renewable Energy Directive. The ratio of net energy imports to gross available energy is the definition of variable x_5_; this indicator represents the share of total energy needs of a country met by energy imports from other countries (net imports = import minus export). The variable x_6_ measures the proportion of the population unable to afford to keep their home adequately warm. The data for the indicator x_6_ are collected as part of the European Union Statistics on Income and Living Conditions (EU-SILC) and this variable monitors the development of poverty and social inclusion in the EU. As the average yearly temperature in a country as well as the share of industrial production can affect the energy consumption, the variables x_7_ and x_8_ were included in the analyzed dataset.

The analysis regarding the set of indicators was conducted in two periods. The first period refers to the year 2010 and the second to 2020. For the analysis of an indicator, the univariate statistical approach was chosen [29–31]. An indicator was described by its minimum, average level, standard deviation, maximum, median and interquartile range. The time span between 2010 and 2020 allowed us to follow the changes of the SDG 7 indicators within the analyzed time span using cumulative relative and absolute changes [32]. The multivariate analysis of the SDG 7 indicators was realized including the average temperature in °C for the period between 1991–2020 and including the average gross value added in industry (except construction) in % of the total GVA for the time span between 2010–2020. Both variables were used to discover the possible association with the SDG 7 indicators and for the building of the cluster analysis model.

Two methods were chosen from among the multivariate techniques, namely the cluster analysis and the principal component analysis, to meet the main aim of the study. The most benefit of the chosen methods are the reduction of the dimension of the analyzed dataset, the solution of collinearity problem among the SDG 7 indicators and the identification of the structures within the analyzed dataset. Cluster analysis (CA) is a useful method for a multivariate analysis that identifies structures within the analyzed dataset [33]. For grouping objects, in our case EU countries, of similar kind into categories different algorithms and methods can be used. These methods represent segmentation analysis that organizes selected variables into meaningful structures, and typical for these methods is that they do not make any distinction between dependent and independent variables [34–38]. Cluster analysis makes it possible to create homogenous groups of objects in such a manner that the objects are similar to one another within a specific cluster, but they are dissimilar to objects in different clusters. In other words, it is expected that the level of association between two objects is maximal if they reside in the same group and is minimal otherwise [39–41]. To compute the distance for different data types, different measures are used and various non-hierarchical or hierarchical methods are applied for identification of which clusters should be joined at each stage. To these methods belong the furthest neighbor method, average linkage method, nearest neighbor method, centroid method, k-means clustering method, Ward’s method [42, 43]. For CA in the study, 27 EU countries were used as objects and six SDG 7 variables including two additional variables were used as indicators. The collinearity of indicators is a usual issue of the multivariate statistical analysis, and therefore it is necessary to find the best possible way how to solve the problem of significantly correlated variables. To solve this issue, principal component analysis (PCA) was selected. PCA is a technique that creates a linear combination of analyzed indicators and belongs to dimensionality reduction methods [44–46].

The principal component analysis generates the same number of components as is the number of originally analyzed variables, but for the next stage of analysis, generally only a few of them are used. The reduced “newly” calculated principals should account for and explain most of the variance in a correlation matrix pattern [47, 48]. The principal component analysis helps identify a minimum number of factors which account for the maximum variance in the original dataset in use, and it means that this technique is suitable for the reduction of a large set of variables to a smaller set of uncorrelated variables named principal components. The first principal component represents the highest variability in the dataset, and each next component accounts for as much of the remaining variability as possible [47, 49, 50].

For the next stage of analysis, it is necessary to involve only the first *m* eigenvectors that explain a predetermined boundary of the total variability. According to recommendation as a common cut-off point can serve, the threshold of 70% of the total variability in the original dataset [51].

The next way to decide about the number of needed principal components is to inspect the scree plot of the principal component analysis that draws the variance explained by each of the components. The number of principal components needed in the next stage of analysis can be determined from the scree plot by the inflection point of the principals and also the Kaiser criterion can be used for selecting all the components for which the eigenvalue is higher than 1 which means that such a corresponding component explains more variance than a single variable [28].

## Results

The analysis of the indicators related to the affordable and clean energy in the EU was done in a few steps. Firstly, the analysis of state and development of the variables was realized, then the correlation analysis made it possible to discover the level of linear relationship of the indicators and the multivariate techniques enabled to uncover the multidimensionality of the SDG 7 indicators.

### Condition and changes of the SDG 7 indicators

The condition of the EU in terms of the set of SDG 7 indicators during the years 2010 and 2020 including two supplementary variables is presented in Tables 1 and 2 while the development of the indicators can be found in Table 3.

**Table 1:** Summary of statistics of the set of indicators in the EU.

Variable	Mean	Std Dev	Minimum	Maximum	Range	Lower Quartile	Median	Upper Quartile
X_1__2010	3.5	1.6	1.6	9.1	7.5	2.4	3.2	4.1
X_2__2010	637.1	233.1	167.0	1084.0	917.0	420.0	665.0	784.0
X_3__2010	6.5	1.6	3.6	9.8	6.3	5.7	6.5	7.6
X_4__2010	16.4	10.8	1.0	46.1	45.1	9.1	13.0	24.2
X_5__2010	56.0	28.2	-16.0	100.6	116.6	38.0	56.9	78.6
X_6__2010	12.0	13.7	0.5	66.5	66.0	3.8	6.8	15.4
X_1__2020	2.9	1.1	1.4	6.3	4.8	2.2	2.6	3.3
X_2__2020	561.5	171.1	204.0	957.0	753.0	416.0	563.0	695.0
X_3__2020	9.5	3.4	5.1	22.4	17.4	7.4	9.1	10.7
X_4__2020	24.4	11.5	10.7	60.1	49.4	16.2	21.2	31.0
X_5__2020	58.0	21.1	10.5	97.6	87.1	42.8	56.6	73.5
X_6__2020	7.8	7.1	1.5	27.5	26.0	2.8	5.7	10.0
X_7__1991–2020	10.3	4.0	2.5	19.6	17.1	8.3	9.9	11.7
X_8__2010–2020	20.0	6.2	6.9	31.3	24.5	16.2	20.0	25.1

**Table 2:** Selected characteristics of the set of indicators in the EU.

Variable	2010	2020	Variable	2010	2020
**X**_**1**_	**Primary energy consumption per capita, TOE per capita**		**X**_**5**_	**Energy import dependency**	
Minimum	1.6	1.4	Minimum	-16.0	10.5
	*(Romania)*	*(Malta)*		*(Denmark)*	*(Estonia)*
Maximum	9.1	6.3	Maximum	100.6	97.6
	*(Luxembourg)*	*(Luxembourg)*		*(Cyprus)*	*(Malta)*
**X**_**2**_	**Final energy consumption in households per capita, KGOE per capita**		**X**_**6**_	**Population unable to keep home adequately warm, %**	
Minimum	167.0	204.0	Minimum	0.5	1.5
	*(Malta)*	*(Malta)*		*(Luxembourg)*	*(Austria)*
Maximum	1084.0	957.0	Maximum	66.5	27.5
	*(Finland)*	*(Finland)*		*(Bulgaria)*	*(Bulgaria)*
**X**_**3**_	**Energy productivity**		**X**_**7**_	**Average temperature in °C, 1991–2020**	
Minimum	3.6	5.1	Minimum	2.5	
	*(Estonia)*	*(Malta)*		*(Finland)*	
Maximum	9.8	22.4	Maximum	19.6	
	*(Ireland)*	*(Ireland)*		*(Cyprus)*	
**X**_**4**_	**Share of renewable energy in gross final energy consumption**		**X**_**8**_	**GVA in industry (except contruction), % of total, 2010–2020**	
Minimum	1.0	10.7	Minimum	6.9	
	*(Malta)*	*(Malta)*		*(Luxembourg)*	
Maximum	46.1	60.1	Maximum	31.3	
	*(Sweden)*	*(Sweden)*		*(Ireland)*	

**Table 3:** Absolute and relative changes of the SDG 7 indicators from 2010 till 2020.

Variable	Relative change, %	Absolute change
**X**_**1**_	-17.1	-0.6
**X**_**2**_	-11.9	-75.6
**X**_**3**_	46.2	3.0
**X**_**4**_	48.8	8.0
**X**_**5**_	3.6	2.0
**X**_**6**_	-35.0	-4.2

The primary energy consumption per capita as well as the final energy consumption in household per capita are both destimulants. In 2010, the lowest primary energy consumption was achieved in Romania followed by Lithuania. In these two countries, the primary energy consumption per capita was lower than 2 tonnes of oil equivalent per capita. The positive change of the indicator *x*_1_ can be rated through a decline of the average value that decreased from 3.5 to 2.9 TOE per capita and it means that the indicator x_1_ dropped by 17.1%. In 2020, altogether in five EU countries the primary energy consumption per capita was lower than 2 tonnes of oil equivalent, namely in Malta, Romania, Greece, Portugal and Croatia. The highest levels of the first indicator were achieved in Luxembourg, where the maximum was as high as 9.09 in 2010 and 6.25 in 2020. The final energy consumption in households per capita was in 2010 when there was an average of 637.1 kilograms of oil equivalent per capita which dropped to 561.5 in 2020 ending with a decrease of nearly 12%. The lowest final energy consumption in household per capita was achieved in Malta while the highest was in Finland in both analyzed years. The energy productivity in purchasing power standard per kilogram of oil equivalent and the share of renewable energy in gross final energy consumption are stimulant variables. These indicators changed in a positive way across the analyzed time span. The indicator x_3_ increased by 46.2% and the indictor x_4_ by 48.8%. In 2010, only in the best country (Ireland) was the energy productivity higher than 9 PPS per kilogram of oil equivalent. The number of countries with a productivity higher than 9 increased to fourteen in 2020 which must be rated very positively. On the other hand, the lowest energy productivity was only 3.6 in Estonia in 2010 and 5.1 in Malta in 2020.

The EU countries did a good job by increasing the share of renewable energy in gross final energy consumption which is an important trend for the future of clean energy production and it is extremely important in a time of energy crisis caused by the war in Ukraine. The average share of renewable energy in gross final energy consumption was 16.4% in 2010 and 24.4% in 2020. The relative variability of the indicator x_4_ was the second highest but the differences between countries are declining over time which is visible from the decline of the coefficient of variation from 65.8% to 47.1%. The best countries regarding the indicator x_4_ were Sweden and Finland. In Sweden, the share of renewable energy in gross final energy consumption was as high as 46.1% in 2010 and it jumped to 60.1% in 2020. Unfortunately, some of the countries do not invest enough effort and capital into the production of renewable energy as their share of the renewable energy in gross final energy consumption was only 1.0% in Malta and 2.9% in Luxembourg at the beginning of the analyzed time period. The share in the two worst countries increased in the case of Malta to 10.7% and in Luxembourg to 11.7% in 2020. In particular, Luxembourg being a country with a very high living standard according to the high values of the gross domestic product per capita, should care more about clean energy production and consumption.

The last two variables from the set of SDG 7 indicators are destimulants. Energy import dependency is crucial especially in times of uncertainty caused by any kind of crises, in times of military conflicts. The energy import dependency was especially high in Luxembourg (97.1%), Malta (99%) and Cyprus (100.6) in 2010 and in these same three countries, it was nearly the same in 2020: Luxembourg (92.5%), Cyprus (93.1%) and Malta (97.6%). In the case of the indicator x_5_, a moderate increase was achieved between 2010 and 2020. The proportion of population unable to keep their home adequately warm dropped in the EU from 12% in 2010 to 7.8% in 2020. The decrease of 35% during the analyzed time period should be rated positively. The decrease of the proportion in the countries with the highest share of population that was unable to keep their homes adequately warm was especially positive. In 2010 in Bulgaria the proportion was extremely high (66.5%), while in Portugal the share reached 30.1% and in Cyprus 27.3%. In 2020, the highest proportion was achieved again in Bulgaria (27.5%) followed by Lithuania (23.1) and Cyprus (20.9%). On the other hand, in some EU countries the share was lower than 2%, namely in Luxembourg (0.5%) and Finland (1.4%) in 2010 and in Austria (1.5%) and Finland (1.8%) in 2020. The territorial and climate differences among the EU countries can be defined by the average temperature in °C. This variable is presented as the average temperature between 1991 and 2020. In Finland and in Sweden, the average temperature was only 2.5 °C and 2.6 °C respectively but in Malta and Cyprus it was 19.6 °C. The next additional variable measures the share of the GVA produced in industry (except the construction industry) as the percentage of the total GVA in a country. The variable x_8_ is calculated as the average proportion of the GVA in industry for the time period between 2010 and 2020. The highest share was typical for Ireland (31.3%), Czechia (30.5%) and Romania (28.0%) while the lowest proportion was achieved in Luxembourg (6.9%).

The relative and absolute changes of the variables between 2010 and 2020 are presented in Table 3 and the changes manifest the direction of shift of affordable and clean energy indicators. A stimulant indicator develops positively in the case of an increase while a destimulant variable shifts positively in the case of a decrease over a time period. The primary energy consumption per capita decreased by 17.1% and the final energy consumption in household per capita dropped by nearly 12% or by 75.6 KGOE per capita during the analyzed time period. As the first two variables are destimulants, the decrease of both must be positively rated. The increase of energy productivity by 46.2% and the jump of the share of renewable energy in gross final energy consumption by 48.8% points to the quite positive direction of these stimulant indictors. The next two variables, i.e., x_5_ and x_6_, are destimulants and so their decrease should be rated positively. Only one of the variables changed in a negative way, namely the energy import dependency ratio that increased by 3.6%. The increase of energy import dependency was not very significant. Much better was the results of changes in the case of the proportion of population which were unable to keep their homes adequately warm, as this indicator declined by 35% or by 4.2 percentage points respectively. The relative and absolute changes presented in Table 3 discovered a positive movement of the SDG 7 indicators except for when it comes to the energy import dependency variable.

## Correlation of the indicators

The indicators that serve to monitor the progress of achieving affordable and clean energy in the EU were used to create a group of countries with similar status of the SDG 7. Before constructing the clusters or groups of EU countries, the correlation analysis was carried out. The purpose of the correlation analysis was to detect whether between the indicators exists a statistically significant linear correlation. In case of a strong linear association, the cluster analysis is done using the uncorrelated principal components instead of the original input variables. The correlation matrix can be found in Tables 4 and 5. The first row indicates the pair correlations level of the Pearson´s correlation coefficient, while the second row represents the probability *p* value of the statistical significance test.

**Table 4:** Correlation matrix of the SDG 7 indicators in 2010.

**Pearson Correlation Coefficients, N = 27**								
**Prob > |r| under H0: Rho = 0**								
	**X**_**1**_ **_2010**	**X**_**2**_ **_2010**	**X**_**3**_ **_2010**	**X**_**4**_ **_2010**	**X**_**5**_ **_2010**	**X**_**6**_ **_2010**	**X**_**7**_	**X**_**8**_
**X**_**1**_ **_2010**	1	0.755	-0.160	-0.006	0.068	-0.490	-0.388	-0.302
		*<* .*0001*	*0*.*425*	*0*.*977*	*0*.*738*	*0*.*009*	*0*.*045*	*0*.*126*
**X**_**2**_ **_2010**	0.755	1	-0.041	0.282	-0.279	-0.643	-0.697	0.080
	*<* .*0001*		*0*.*839*	*0*.*154*	*0*.*159*	*0*.*000*	*<* .*0001*	*0*.*693*
**X**_**3**_ **_2010**	-0.160	-0.041	1	-0.050	0.227	-0.046	0.231	0.016
	*0*.*425*	*0*.*839*		*0*.*805*	*0*.*254*	*0*.*821*	*0*.*245*	*0*.*939*
**X**_**4**_ **_2010**	-0.006	0.282	-0.050	1	-0.422	-0.063	-0.621	0.184
	*0*.*977*	*0*.*154*	*0*.*805*		*0*.*029*	*0*.*754*	*0*.*001*	*0*.*359*
**X**_**5**_ **_2010**	0.068	-0.279	0.227	-0.422	1	0.111	0.526	-0.391
	*0*.*738*	*0*.*159*	*0*.*254*	*0*.*029*		*0*.*583*	*0*.*005*	*0*.*044*
**X**_**6**_ **_2010**	-0.490	-0.643	-0.046	-0.063	0.111	1	0.349	-0.030
	*0*.*009*	*0*.*000*	*0*.*821*	*0*.*754*	*0*.*583*		*0*.*074*	*0*.*883*
**X**_**7**_	-0.388	-0.697	0.231	-0.621	0.526	0.349	1	-0.464
	*0*.*045*	*<* .*0001*	*0*.*245*	*0*.*001*	*0*.*005*	*0*.*074*		*0*.*015*
**X**_**8**_	-0.302	0.080	0.016	0.184	-0.391	-0.030	-0.464	1
	*0*.*126*	*0*.*693*	*0*.*939*	*0*.*359*	*0*.*044*	*0*.*883*	*0*.*015*	

*p* values in italics

**Table 5:** Correlation matrix of the SDG 7 indicators in 2020.

**Pearson Correlation Coefficients, N = 27**								
**Prob > |r| under H0: Rho = 0**								
	**X**_**1**_ **_2020**	**X**_**2**_ **_2020**	**X**_**3**_ **_2020**	**X**_**4**_ **_2020**	**X**_**5**_ **_2020**	**X**_**6**_ **_2020**	**X**_**7**_	**X**_**8**_
**X**_**1**_ **_2020**	1	0.768	-0.063	0.132	-0.042	-0.442	-0.530	-0.156
		*<* .*0001*	*0*.*754*	*0*.*512*	*0*.*834*	*0*.*021*	*0*.*004*	*0*.*438*
**X**_**2**_ **_2020**	0.768	1	0.109	0.317	-0.316	-0.630	-0.761	0.184
	*<* .*0001*		*0*.*590*	*0*.*108*	*0*.*109*	*0*.*000*	*<* .*0001*	*0*.*359*
**X**_**3**_ **_2020**	-0.063	0.109	1	-0.098	0.146	-0.093	0.015	0.226
	*0*.*754*	*0*.*590*		*0*.*627*	*0*.*469*	*0*.*645*	*0*.*941*	*0*.*258*
**X**_**4**_ **_2020**	0.132	0.317	-0.098	1	-0.506	-0.083	-0.591	0.057
	*0*.*512*	*0*.*108*	*0*.*627*		*0*.*007*	*0*.*680*	*0*.*001*	*0*.*777*
**X**_**5**_ **_2020**	-0.042	-0.316	0.146	-0.506	1	0.265	0.650	-0.546
	*0*.*834*	*0*.*109*	*0*.*469*	*0*.*007*		*0*.*182*	*0*.*000*	*0*.*003*
**X**_**6**_ **_2020**	-0.442	-0.630	-0.093	-0.083	0.265	1	0.469	-0.220
	*0*.*021*	*0*.*000*	*0*.*645*	*0*.*680*	*0*.*182*		*0*.*014*	*0*.*270*
**X**_**7**_	-0.530	-0.761	0.015	-0.591	0.650	0.469	1	-0.464
	*0*.*004*	*<* .*0001*	*0*.*941*	*0*.*001*	*0*.*000*	*0*.*014*		*0*.*015*
**X**_**8**_	-0.156	0.184	0.226	0.057	-0.546	-0.220	-0.464	1
	*0*.*438*	*0*.*359*	*0*.*258*	*0*.*777*	*0*.*003*	*0*.*270*	*0*.*015*	

*p* values in italics

**Fig 1:**
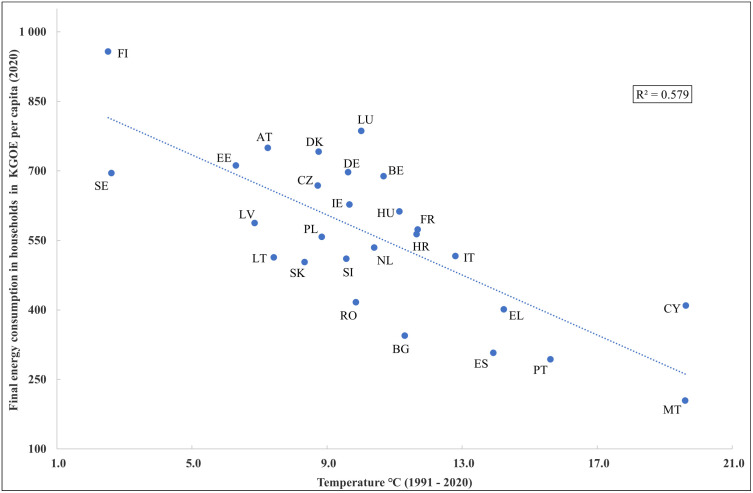
Association between temperature and final energy consumption in households per capita.

The correlation between the indictors x_1_ and x_2_ was quite strong and positive as this correlation reached 0.755 in 2010 and 0.768 in 2020. A strong and positive association between primary energy consumption per capita and final energy consumption in household per capital was expected and was also confirmed using the correlation analysis. The indicator x_1_ was strongly correlated also with the indicator x_6_. The variable x_2_ was strongly correlated not only with the indicator x_1_ but also with the variables x_6_ and x_7_. The association between variables x_2_ and x_7_ was negative and very high, as it reached -0.697 in 2010 and -0.761 in 2020. This very strong and negative correlation between final energy consumption in households per capita and the average temperature between the years 1991–2020 points to the fact that in countries with a low average temperature, a higher final energy consumption per capita is expected, while in countries with a higher average temperature, a lower final energy consumption in households per capita is a reality (see Fig 1).

### Cluster analysis

The cluster analysis allowed us to group countries with a similar status regarding the sustainable indicators of affordable and clean energy. The analysis conducted in two periods, namely in 2010 and 2020, enabled us to follow the changes of a country’s position. Countries that joined together into a specific cluster could commonly act and try to find the best way to continue to achieve the ambitious goals of the sustainability issue.

#### Cluster analysis in 2010

**Fig 2:**
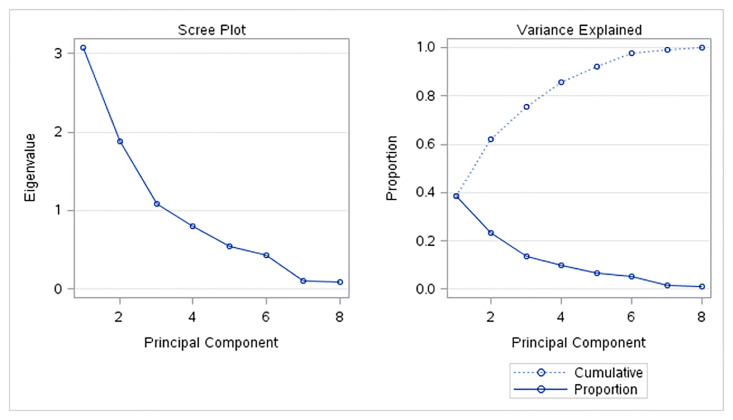
Principal components analysis in 2010.

The correlation analysis discovered a very strong or strong correlation between some pairs of the SDG 7 indicators and therefore the cluster analysis was done using the most important uncorrelated principals. For the cluster analysis in the year 2010, only the first three most important principles were used. These uncorrelated first three principal components explain more than 75% of the variation of the original dataset (see Fig 2).

**Fig 3:**
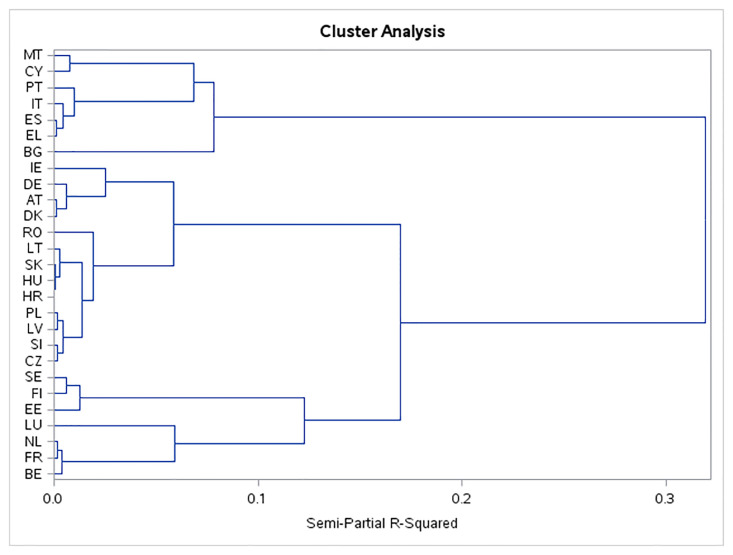
Dendrogram tree of the cluster analysis in 2010.

During the year 2010, according to the cluster tree presented in Fig 3, the EU countries were split into five relatively isolated clusters. The clusters separated the twenty-seven EU member states into three clusters with a frequency of 3, 4 and 6 countries, one cluster with a higher frequency of 13 objects and only one cluster that contains a solo country, namely Bulgaria.

Table 6 represents the cluster centroids, i.e., the main features of the SDG 7 indicators of the EU countries classified into a specific cluster in 2010.

**Table 6:** Cluster centroids of the EU countries regarding the SDG 7 in 2010.

Clusters of the EU countries, 2010	*Cluster 1*	*Cluster 2*	*Cluster 3*	*Cluster 4*	*Cluster 5*
Number of countries	*6*	*1*	*13*	*3*	*4*
**X**_**1**_ **_2010**	2.6	2.4	2.9	5.3	5.6
**X**_**2**_ **_2010**	372.5	303.0	660.1	912.7	836.5
**X**_**3**_ **_2010**	7.3	4.7	6.8	4.5	6.1
**X**_**4**_ **_2010**	11.4	13.9	17.8	34.3	6.4
**X**_**5**_ **_2010**	83.8	40.1	47.3	33.8	63.1
**X**_**6**_ **_2010**	17.7	66.5	10.0	2.2	3.5
**X**_**7**_	16.0	11.3	9.1	3.8	10.7
**X**_**8**_	14.3	22.3	24.3	20.3	13.4

*Cluster 1 (Malta*, *Cyprus*, *Portugal*, *Italy*, *Spain*, *Greece)*. The Southern European countries of the EU were joined together in the first cluster. It was typical for these countries to have the highest average temperature among the clusters. This high average temperature was associated with the lowest primary energy consumption and second highest final energy consumption in household per capita. These results are consistent with the correlation analysis that discovered a strong and negative association between the average temperature and energy consumption indicators. As a threat for these countries, we could see that the highest percentage of energy import dependency was in this group of countries being as high as 83.8%. The maximal value of energy productivity among these European countries must be rated quite positively. The countries united into the first cluster should put more effort towards renewable energy as the share of the renewable energy in gross final energy consumption was the second lowest.

*Cluster 2 (Bulgaria)*. The only solo cluster in 2010 consisted of a less developed country in the EU that joined the EU in 2007. For the country, having a low GDP per capita and a lower living standard could be the reason for having the lowest final energy consumption in households per capita and the lowest primary energy consumption per capita. The minimal values of the indicator x_1_ and x_2_ can also be the result the average temperature in 1991–2020 was the second lowest among the clusters. A second possible explanation for the minimal level of final energy consumption in households per capita is that it was caused by the extremely high share of population unable to keep their home adequately warm; the indicator x_6_ in Bulgaria reached a maximum level of 66.5%.

*Cluster 3 (Ireland*, *Germany*, *Austria*, *Denmark*, *Romania*, *Lithuania*, *Slovakia*, *Hungary*, *Croatia*, *Poland*, *Latvia*, *Slovenia*, *Czechia)*. Most of the EU countries were joined together into the third cluster. Nine countries belong to the “newer” member states and only four countries belong to the “older” EU members. These countries reached a maximal value of the percentage of GVA in industry while according to indicators x_1_, x_2_, x_5_, x_6_ and x_7_ they are in the middle among the clusters. It should be positively rated as it saw the second highest energy productivity and also the second highest share of the renewable energy in gross final energy consumption in this group of countries.

*Cluster 4 (Sweden*, *Finland*, *Estonia)*. Typical for Northern European countries is the low average temperature in °C that was as low as 3.8 °C compared to 16 °C in the first cluster. The very low temperature was associated with the highest average final energy consumption in households per capita that achieved 912.7 KGOE per capita compared with only 372.5 KGOE in the first cluster. A common positive sign of these countries was the highest share of renewable energy in gross final energy consumption that reached a proportion of 34.3%. As the highest final energy consumption in household per capita and the second highest primary energy consumption in these countries is associated with the highest share of renewable energy in consumption, it gave Sweden, Finland, and Estonia a quite good position within the sustainability effort of the EU countries in the area of affordable and clean energy. The high living standard in the North-European countries resulted in the minimal value of the proportion of population unable to keep their homes adequately warm. Minimal was also the energy import dependency indicator that creates a good position for these countries in the area of energy independency.

*Cluster 5 (Luxembourg*, *the Netherland*, *France*, *Belgium)*. The countries that were joined together in cluster 5 achieved the highest primary energy consumption per capita and the second highest final energy consumption in households per capita and it means that these countries belong to EU member states with a high energy demand per capita. Unfortunately, these well-developed countries had on average the lowest share of renewable energy in gross final energy consumption. A combination of both conditions, i.e., high energy consumption and a very low proportion of the renewable energy consumption are a negative sign for affordable and clean energy in societies. The population of these well-developed countries rarely have problems with their inability to keep their homes adequately warm. The energy dependency of these group of countries was higher than 63% which was among the second highest level among countries.

#### Cluster analysis in 2020

**Fig 4:**
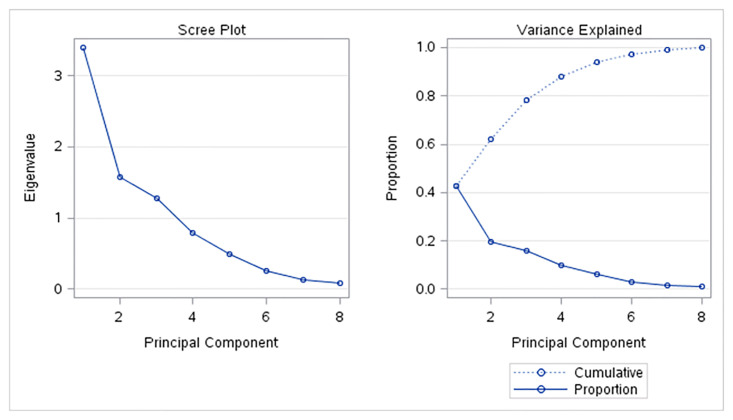
Principal components analysis in 2020.

**Fig 5:**
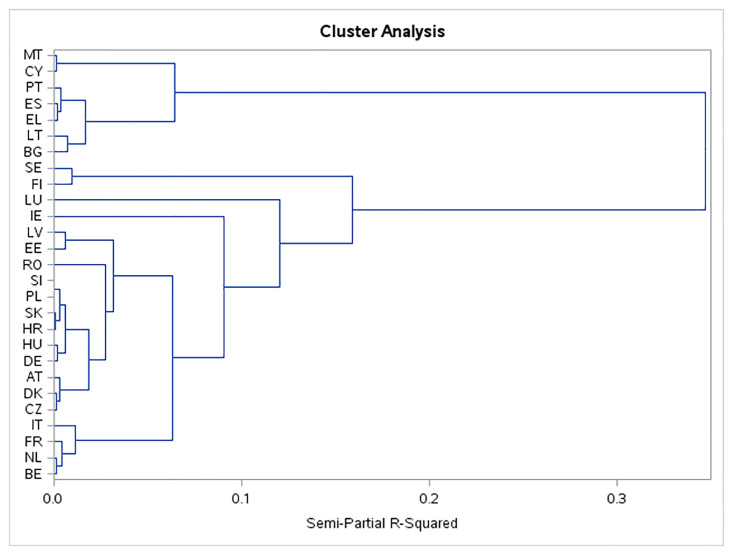
Dendrogram tree of the cluster analysis in 2020.

Similarly, as in the year 2020, since some of the analyzed variables were very strongly or strongly correlated, the cluster analysis was done using the most important principals. For the cluster analysis in the year 2020, again the first three most important principles were used. Altogether, these three uncorrelated principal components explain more than 78% of the variation of the original indicators (Fig 4). The twenty-seven EU member countries were in 2020 classified into seven clusters (Fig 5).

Table 7 contains the cluster centroid of the EU countries classified into a specific cluster in 2020.

**Table 7:** Cluster centroids of the EU countries regarding the SDG 7 in 2020.

Clusters of the EU countries, 2020	*Cluster 1*	*Cluster 2*	*Cluster 3*	*Cluster 4*	*Cluster 5*	*Cluster 6*	*Cluster 7*
Number of countries	*2*	*5*	*2*	*1*	*1*	*12*	*4*
**X**_**1**_ **_2020**	2.0	2.1	4.7	6.3	2.7	2.7	3.1
**X**_**2**_ **_2020**	306.5	371.6	826.0	786.0	627.0	609.5	577.8
**X**_**3**_ **_2020**	7.1	9.1	6.9	12.5	22.4	9.4	9.1
**X**_**4**_ **_2020**	13.8	25.4	52.0	11.7	16.2	25.4	16.6
**X**_**5**_ **_2020**	95.3	65.5	37.8	92.5	71.3	45.4	66.0
**X**_**6**_ **_2020**	14.1	19.2	2.3	3.6	3.3	4.5	5.4
**X**_**7**_	19.6	12.5	2.6	10.0	9.7	8.9	11.4
**X**_**8**_	9.6	18.5	19.9	6.9	31.3	23.6	16.4

*Cluster 1 (Malta*, *Cyprus)*. Two Mediterranean islands joined together to create the first cluster in 2020. Typical for these southern European islands was the extremely high average temperature that was as high as 19.6 °C. These countries were in a common cluster with countries like Portugal, Italy, Spain and Greece in 2010. According to the findings of the association between the pairs of analyzed variables, it was expected that in countries with a high average temperature during the period between 1991–2020, a low energy consumption per capita is expected and these findings were supported as result of the cluster analyses. Malta and Cyprus reached the highest average temperature but they were faced with quite minimal primary energy consumption per capita and also with the minimal final energy consumption in households per capita. On the other hand, in these countries a long road towards achieving renewable energy is a reality. Only 13.8% of the gross final energy consumption comes from renewable sources. The energy import dependency of these island countries was the highest among the clusters and it was higher than 95%. It will be extremely important for the economies of both Mediterranean countries to invest into renewable solar energy and to increase the production and consumption of the renewable energy sources which could be generated from sunlight directly.

*Cluster 2 (Portugal*, *Spain*, *Greece*, *Lithuania*, *Bulgaria)*. Southern European countries created a cluster together with Bulgaria and Lithuania. In these countries, the average temperature was the second highest that resulted in the second lowest primary energy consumption per capita and second lowest final energy consumption in households per capita. It is positive that the share of the renewable energy was about 25.4% compared to only 13.8% for countries in the first cluster. On the other hand, it was not positive that the proportion of the population unable to keep their home adequately warm was the highest among all clusters. The main reason as to why Malta and Cyprus were not joined together in 2020 with other southern European countries was their extremely high energy import dependency, very low share of the renewable energy, as well as lower energy productivity.

*Cluster 3 (Sweden*, *Finland)*. The countries of Northern Europe were joined together in the year 2010 and in 2020 as these territorially close EU member states created a common cluster again. These countries had the lowest average temperature that was associated with the highest final energy consumption in household per capita and the second highest primary energy consumption per capita. The population of Sweden and Finland are able to keep their homes adequately warm despite the very low temperatures in both countries as the indicator x_6_ was the most minimal when considering all of the clusters. The positive movement of both countries toward affordable and clean energy is visible from their highest share of renewable energy in gross final energy consumption as this share was as high as 52%. The strong negative correlation between variables x_4_ and x_5_ can be noticeable from the combination of the highest share of renewable energy and the lowest energy import dependency of countries that were joined together into cluster three.

*Cluster 4 (Luxembourg)*. Two solo clusters were created in the year 2020 compared with only one solo cluster in 2010. Cluster four represents Luxembourg as a very developed country with a high living standard. This cluster reached the lowest share of renewable energy when it comes to gross final energy consumption and the highest primary energy consumption per capita. The final energy consumption in households per capita was the second largest. The combination of the largest level of indicator x_1_ and second largest value of the indicator x_2_ with the lowest share of renewable energy in energy consumption creates a bad view of Luxembourg in the effort to do more for climate and more for clean energy. As a result of the previously mentioned situation of Luxembourg, its economy is strongly dependent on energy imports. The indicator x_5_ that describes a country´s energy import dependency was higher than 92%.

*Cluster 5 (Ireland)*. The highest energy productivity among the clusters was achieved in cluster 5, i.e., in Ireland. Highest was also the share of the GVA created in industry. It must be viewed positively that there was a very small proportion of population that is unable to keep their home adequately warm (3.3%). Energy import dependency that reached 71.3% could be reduced by increasing the share of renewable energy in gross final energy consumption that was only 16.2%.

*Cluster 6 (Latvia*, *Estonia*, *Romania*, *Slovenia*, *Poland*, *Slovakia*, *Croatia*, *Hungary*, *Germany*, *Austria*, *Denmark*, *Czechia)*. Most of the new member states joined together in cluster 6 with Germany, Austria and Denmark. The cluster is, according to some of the analysed indicators, in the middle among the clusters. The middle position was typical for the variables x_1_, x_2_, x_3_, x_6_ while the indicator of GVA in industry was the second highest. The average temperature in this cluster was only 8.9 °C and therefore a higher energy consumption was expected. It is positive that this cluster had a higher share of renewable energy in gross final energy consumption (24.4%) and a lower energy import dependency (45.4%).

*Cluster 7 (Italy*, *France*, *the Netherlands*, *Belgium)*. Countries of Western Europe and Italy created a common cluster that had a bit worse position than cluster 6. This was true for the indicators x_1_, x_3_, x_4_, x_5_, x_6_ but just as in the prior cluster, cluster 7 can be rated as being in the “middle” with the levels of the variables being neither good nor bad.

## Discussion

The issue of sustainable development has been a frequently discussed topic in recent years, both at the national and international level. The 2030 Agenda for Sustainable Development is one of the basic strategies that goes beyond the framework of the EU and establishes 17 SDGs in various areas, which present a common plan to support countries in their efforts to reconcile economic growth with sustainability [52]. Monitoring and fulfilling goals through several indicators contribute to sustainable development in the future period.

This study is focused on the area of affordable and clean energy (SDG 7). It includes a description of 6 indicators forming the basis of SDG 7 and 2 additional indicators, a comparison of the achieved results with respect to the size of the monitored indicators of the EU27. For the development of mutual relations between individual indicators, a correlation matrix of the SDG 7 indicators in 2010 and 2020 was created. Any ranking and grouping of European countries in context of SDGs may differ depending on the assessment method adopted. Several studies use the cluster analysis [20, 53]. Also, we used the cluster analysis to unveil latent association structures. Based on it, member countries with similar achieved results for individual indicators were divided into five clusters in 2010 and seven clusters in 2020.

Based on the performed cluster analysis, we noted the largest dynamic shift between individual clusters was in the case of Italy, Luxembourg and Estonia. The most positive shift occurred in Italy due to a significant shift in four indicators. A decrease in the indicator primary energy consumption per capita by 20.9%, a positive decrease in the indicator population unable to keep their home adequately warm by 28.4%, an increase in energy productivity by 32% and an increase in the share of renewable energy in gross final energy consumption by 56.3%. Thanks to the increase in the last-mentioned indicator, the country managed to fulfil the target of the Europe 2020 strategy in this area since 2014. Dello Strologo et al. [54] also share the opinion that Italy is well on its way to progress and is also converging towards achieving the goals of the 2030 Agenda in the field of SDG 7.

In Luxembourg, exactly the opposite shift occurred within the clusters. In 2010, the country had the highest rate of primary energy consumption per capita among member countries and despite a 31.2% drop in this consumption, the value of the indicator is still the highest in the EU27. There was a positive increase in the indicator energy productivity by 67.6% and also the increase in the indicator expressing the share of renewable energy in gross final energy consumption by 310% between 2010 and 2020. Despite such a significant increase, it was the second-lowest increase from the member countries. The results in 2020 are significantly influenced by the starting value from 2010. Even though great progress has occurred in the monitored 10 years, it still appears to be insufficient at the established pace. Another problem of Luxembourg is the energy import dependency, which is one of the highest and in 10 years there has been only a slight reduction in this dependence. Cheba and Bak [55] also reached similar conclusions. According to the results of Simionescu et al. [56], this country diverges from the climate/energy targets because of the potential relationship between the indicators the share of renewable energy sources in electricity and the share of real GDP per capita.

Estonia is also among the countries that have seen a more significant shift within the clusters. This worsened its position. The reason for its exclusion from the cluster from Finland and Sweden is the lowest value of the indicator energy import dependency (10%) in the EU. The second-highest increase in the energy productivity indicator by 96.9% during the years 2010 and 2020 was positively evaluated. Despite this fact, the country was fifth from the end among the member countries in this indicator. On the other hand, thanks to the increase in the share of renewable energy in gross final energy consumption, Estonia has been achieving the target of the Europe 2020 strategy since 2011. A study by Rybak [53] discusses reducing dependence on energy imports and also increasing the use of renewable energy sources in Estonia.

Next, according to the analyse, it can be concluded that the southern countries of the EU are characterized by a higher average temperature compared to other member countries, which positively contributes to a lower rate of primary energy consumption per capita and the final energy consumption in households per capita. On the other hand, countries such as Malta and Cyprus, which formed a separate cluster in 2020, had a problem with extremely high energy import dependency, lower energy productivity and a very low share of renewable energy (13.8%, which is very far from the required target value in 2030 Agenda at a level of 32%). Vavrek and Chovancová [57] wrote that Cyprus belongs among the lagging EU countries from the point of view of energy performance. Likewise, Tutak et al. [17] confirmed low levels of energy sustainability of new member states (incl. Cyprus and Malta). In contrast to their conclusions, the opposite views can also be encountered. For example, Guzowska and Kryk [58] state that new member countries Latvia, Malta and Cyprus were most effective in approaching the targets in environmental area in 2018. According to Firoiu et al. [18], it is possible to expect a higher energy import dependency in the case of small but also island states such as Cyprus and Malta. At the same time, these countries should take advantage of their geographical location in increasing the share of renewable energy in gross final energy consumption and energy productivity.

In contrast, the northern countries of the EU such as Sweden and Finland, are characterized by the lowest average temperature during the period between 1991–2020, which led to the highest final energy consumption and relatively high per capita primary energy consumption in 2020. However, achieving the best results in the use of renewable energy sources (52%, countries significantly exceed the required target value) is a positive. Guzowska and Kryk [58] reached similar conclusions in their paper, according to which Sweden’s high position is related to the long-term implementation of a very precise and purposeful environmental policy. Since the 1970s, the country has increased the emphasis on renewable energy sources and gradually reduced the share of fossil fuel use. The most numerous cluster in surveyed years was formed by mostly "newer" member countries of the EU. Unlike older countries, they are characterized by average values in most monitored indicators and a relatively lower dependence on energy imports, which can be evaluated positively.

In our study, we confirmed that the member states of the EU differ significantly in the promotion and achievement of SDG 7. Developments and circumstances in recent years have highlighted these differences even more. These results are consistent with several studies [18, 53, 55]. The sources of these differences between EU member countries can be found, for example, in the different composition of the energy mix, in different energy productivity, in the different degree of dependence on energy imports, but also in differences related to the population which according to the poverty indicator, cannot heat itself sufficiently. The identified deepening diversity between countries significantly threatens the successful achievement of SDG 7 within the set deadline.

## Conclusion and policy recommendations

In view of the growing emphasis placed by the EU on the area of sustainable development and the corresponding goals of the 2030 Agenda, our research was based on an analysis of the level of development of EU member countries in relation to SDG 7. Therefore, the aim of the study was to analyze changes in the development of sustainable indicators set of affordable and clean energy in relation to SDG 7 in the EU, using cluster analysis to identify the differences and compare changes in the grouping of countries into clusters in the two monitored years (2010 and 2020). In the article, we used univariate and multivariate analysis, comparison and identification of associations between selected indicators to monitor the membership of countries in clusters and their movement within clusters.

The results confirmed a positive development of indicators such as primary energy consumption per capita, final energy consumption in households per capita, energy productivity, share of renewable energy in gross final energy consumption and population unable to keep their home adequately warm. On the other hand, energy import dependency has turned out to be problematic.

At the same time, we identified a connection between the selected indicators, namely the relationships between: primary energy consumption per capita and final energy consumption in households per capita; final energy consumption in households per capita and the indicator of population unable to keep their home adequately warm; final energy consumption in households per capita and average temperature; and also primary energy consumption per capita and indicator of population unable to keep their home adequately warm.

The selection of indicators corresponded to the set of indicators identified as key by Eurostat. The indicator of population unable to keep their home adequately warm seems questionable to the authors. Its practical significance is low because the average duration of the heating season is incomparable in the northern and southern regions of the EU and at the same time, cannot be influenced by the countries themselves. This is also confirmed by its strong correlation dependence with the average temperature indicator.

The cluster analysis pointed to significant differences between EU countries as for affordable and clean energy. Despite the fact that energy has an irreplaceable place in the development of the European economy, the individual member states of the EU apply their own, often different, energy policies. Sweden and Finland can be marked as leaders, which as countries with high energy consumption are applying renewable energy sources to a greater extent, by which they make a positive movement toward affordable and clean energy. The mentioned contexts confirm that one of the ways to get closer to achieving SDG 7 is to increase the emphasis on the use and development of renewable energy sources. However, Luxembourg as a well-developed country had on average the lowest share of renewable energy in gross final energy consumption. The combination of high energy consumption and very low proportion of the renewable energy consumption are a negative sign for the affordable and clean energy in societies. The pandemic caused by the disease COVID-19 significantly affected the development of total energy consumption. Various measures and restrictions that were adopted in individual countries, especially in the area of public life and overall lower economic activity, has led to a decrease in energy consumption in 2020. This resulted in an increase in the indicator expressing the share of renewable sources in gross final energy consumption [24]. Only the next few years will show (e.g. analyses in 5 years) real progress in this area, i.e. whether the increase in the share of renewable energy in gross final energy consumption is the result of appropriately taken steps towards a green economy or just a secondary consequence of various restrictions that were adopted by economies in the area of production, provision of services and overall limited activity of the population due to the pandemic caused by COVID-19. This is also one of the reasons why it is necessary to pay more attention to this topic and to abstract from the current (possible distortion) results. However, there are many factors, which will have an impact on the achieved results in the field of renewable energy sources, as well as SDG 7 as a whole. In this context there are relatively rapid climate changes, when the war in Ukraine causes tension and uncertainty in the geopolitical situation, as well as when the energy crisis significantly negatively affects the functioning of individual economic entities, problems related to access to energy come to the fore even more. In these contexts, the importance of renewable energy sources as an alternative source to coal, gas and oil is growing. They also promote energy security and contribute positively to sustainable development worldwide [59]. In addition, the high energy import dependency of the EU is causing vulnerability to supply shocks, which has been fully manifested in the current form of the energy crisis that Europe is facing [60, 61]. Achieving SDG 7 represents an important challenge that requires great efforts and commitment from companies, governments and citizens at national and international levels. This is another reason why Europe must now redouble its efforts to become energy self-sufficient and focus directly on renewable energy sources.

More pragmatic approaches to the introduction of environmental innovations are needed, especially in industrially oriented economies. This requires allocating a part of budgets and resources for research activities aimed at innovations and technologies in the field of clean energy, supporting the use of environmentally friendly equipment and machines, for example through the provision of tax breaks and incentives. This also corresponds with the recommendations of [62].

In order to achieve commitments and goals by 2030, international cooperation must play a leading role in energy policies, support the exchange of experience and knowledge. The public sector plays an important role here, which can make available resources from EU funds, incentives and subsidies. It thus supports the activities of the private sector towards affordable and clean energy.

A potential limitation of this research is represented by the availability of the data and the methodological framework. The limitation of this research is the comparability of the achieved results, which is limited to studies working with a sample of EU countries. The reason is the choice of indicators and specific data, which are adjusted by Eurostat itself, which limits the comparison with other countries at the global level.

In this study, we have provided an insight into the issue of environmental sustainability through the perspective of SDG 7, following the two original targets of the Europe 2020 strategy in the area of climate change and energy. The findings of this research could generate future research directions, expand the set of variables used, for example to include indicators related to SDG 13 in addition to the selected indicators. This is, for example, the consideration of greenhouse gas emissions, because several studies [63–66] confirm the positive relationship between CO2 emissions and economic growth.

## Abbreviations

CA: Cluster analysis
EU: European Union
EU-SILC: European Union—Statistics on income and living conditions
GDP: Gross domestic product
GVA: Gross value added
KGOE: Kilograms of oil equivalent
PCA: Principal component analysis
PPS: Purchasing power standard
SDG: Sustainable Development Goal
TOE: Tonnes of oil equivalent

**EU Country codes:**
AT: Austria
BE: Belgium
BG: Bulgaria
CY: Cyprus
CZ: Czech Republic
DE: Germany
DK: Denmark
EE: Estonia
EL: Greece
ES: Spain
FI: Finland
FR: France
HR: Croatia
HU: Hungary
IE: Ireland
IT: Italy
LT: Lithuania
LU: Luxembourg
LV: Latvia
MT: Malta
NL: Netherlands
PL: Poland
PT: Portugal
RO: Romania
SE: Sweden
SI: Slovenia
SK: Slovakia
